# A randomized, controlled trial comparing local infiltration analgesia with epidural infusion for total knee arthroplasty

**DOI:** 10.3109/17453674.2010.519165

**Published:** 2010-10-08

**Authors:** Karen V Andersen, Marie Bak, Birgitte V Christensen, Jørgen Harazuk, Niels A Pedersen, Kjeld Søballe

**Affiliations:** ^1^Department of Orthopedic Surgery, Aarhus University Hospital, Aarhus; ^2^Department of Anesthesiology; ^3^Department of Orthopedic Surgery, Glostrup Hospital, Glostrup; ^4^The Lundbeck Foundation for Fast Track Hip and Knee Surgery, Copenhagen, Denmark

## Abstract

**Background:**

There have been few studies describing wound infiltration with additional intraarticular administration of multimodal analgesia for total knee arthroplasty (TKA). In this study, we assessed the efficacy of wound infiltration combined with intraarticular regional analgesia with epidural infusion on analgesic requirements and postoperative pain after TKA.

**Methods:**

40 consecutive patients undergoing elective, primary TKA were randomized into 2 groups to receive either (1) intraoperative wound infiltration with 150 mL ropivacaine (2 mg/mL), 1 mL ketorolac (30 mg/mL), and 0.5 mL epinephrine (1 mg/mL) (total volume 152 mL) combined with intraarticular infusion (4 mL/h) of 190 mL ropivacaine (2 mg/mL) plus 2 mL ketorolac (30 mg/mL) (group A), or (2) epidural infusion (4 mL/h) of 192 mL ropivacaine (2 mg/mL) combined with 6 intravenous administrations of 0.5 mL ketorolac (30 mg/mL) for 48 h postoperatively (group E). For rescue analgesia, intravenous patient-controlled-analgesia (PCA) morphine was used.

Morphine consumption, intensity of knee pain (0–100 mm visual analog scale), and side effects were recorded. Length of stay and corrected length of stay were also recorded (the day-patients fulfilled discharge criteria).

**Results:**

The median cumulated morphine consumption, pain scores at rest, and pain scores during mobilization were reduced in group A compared to group E. Corrected length of stay was reduced by 25% in group A compared to group E.

**Interpretation:**

Peri- and intraarticular analgesia with multimodal drugs provided superior pain relief and reduced morphine consumption compared with continuous epidural infusion with ropivacaine combined with intravenous ketorolac after TKA.

Total knee arthroplasty (TKA) usually results in severe postoperative pain. Continuous epidural infusion with a local anesthetic is a standard regime for postoperative analgesia after TKA. Epidural analgesia and also peripheral nerve block analgesia have been shown to reduce opioid consumption compared with intravenous patient-controlled analgesia (PCA). Even though both modalities reduce the occurrence of the well-known side effects of opioid drugs, they involve extra equipment and are associated with substantial side effects (Choi et al. 2003, Davies et al. 2004, Boezaart 2006). Wound infiltration with multimodal analgesia has been a controversial issue for many years (Dahl et al. 1994). Different modes of perioperative analgesia either without or combined with intraarticular infusion or bolus injection(s) for both TKA and total hip arthroplasty have been described (Bianconi et al. 2003, Rasmussen et al. 2004, Reilly et al. 2005, Andersen et al. 2007a, b). Only a few studies have described high-volume peri- and intraarticular analgesia for TKA (Busch et al. 2006, Vendittoli et al. 2006, Toftdahl et al. 2007). The hypothesis in our trial was that wound infiltration and intraarticular infusion of ropivacaine and ketorolac would reduce opioid consumption during the active treatment period (0–48 h postoperatively) after TKA compared to epidural infusion of ropivacaine and intravenous ketorolac. Primary outcome was 48-h opioid use. Secondary outcomes included pain at rest and during mobilization, side effects, length of hospital stay (LOS), and corrected length of stay (the day-patients fulfilled discharge criteria).

## Patients and methods

With the approval of the Ethics Committee of the County of Copenhagen (KA-20060134) and the Danish Medicines Agency (EudraCT.no 2006-004638-33) and after obtaining written informed consent, 40 patients >18 years of age undergoing elective, unilateral, primary TKA were enrolled in this randomized study. The study fulfilled the requirements of the Helsinki Declaration, and was conducted in accordance with GCP-ICH guidelines and monitored by the GCP unit at Copenhagen University Hospital. It was also registered at ClinicalTrial.gov (NCT00421967) and at the Danish Data Protection Agency.

Exclusion criteria were hypersensitivity to study drugs, contraindications to spinal anesthesia and epidural analgesia, regular narcotic use, general anesthesia, inability to communicate in Danish, drug-treated diabetes, neuropathic pain or sensory disorders in the leg to be operated on, rheumatoid arthritis, pregnancy, severe obesity (BMI > 40), and treatment with tricyclic antidepressants, antiepileptic drugs, and/or antacids. Secondary exclusion criteria were failure of spinal anesthesia and reoperation or trauma to the knee within the first 48 h postoperatively.

Eligible patients were randomized before surgery by using a block size of 8 with a ratio of 1:1 according to a computer-generated sequence, and each patient was assigned by opening a sealed envelope to receive either (1) wound infiltration combined with intraarticular infusion (group A) or (2) epidural infusion and intravenous treatment (group E).

Patients did not receive any premedication. To reduce blood loss, tranexamic acid (10 mg/kg) was given at the beginning of surgery and repeated 3 h postoperatively. No surgical drains or bladder catheters were used in any of the patients. Spinal anesthesia was induced at the L2-3 level by using a 27-gauge spinal needle with a dose of 3 mL plain bupivacaine (5 mg/mL).

In group A, at the end of surgery a mixture of 150 mL ropivacaine (2 mg/mL) and 1 mL ketorolac (30 mg/mL) was prepared. 50 mL of the solution was loaded into one 50-mL syringe. 0.5 mL epinephrine (1 mg/mL) was added to the remaining 101 mL, giving a total volume of 102 mL, and loaded into two 50-mL syringes, which the surgeon used to infiltrate below the capsule, muscles, and subcutaneous tissues. The syringe without epinephrine was used to infiltrate skin and subcutis in equal proportions along the whole length of the wound. A multi-hole epidural catheter was placed with the tip in the joint. The catheter was tunneled 8–10 cm subcutaneously and connected to an infusion pump delivering a continuous infusion (flow-rate: 4 mL/h for 48 h) of 190 mL ropivacaine (2 mg/mL) at 8 mg/h plus 2 mL ketorolac (30 mg/mL) at 1.25 mg/h.

In group E, a combined spinal-epidural technique was used. On regression of the spinal block, a test dose of 3 mL lidocaine-adrenalin (20 mg/mL and 5 μg/mL) was given to confirm extradural positioning and this was followed by a bolus of 7 mL ropivacaine (2 mg/mL). An infusion pump was connected with a flow rate of 4 mL/h ropivacaine (2 mg/mL, 8 mg/h) for 48 h postoperatively. 0.5 mL intravenous ketorolac (30 mg/mL) was given perioperatively and 3, 20, 28, 34, and 42 h postoperatively (in a total volume of 3 mL). Both groups (E and A) received a total of 90 mg ketorolac during the active treatment period.

For rescue analgesia, intravenous PCA morphine (concentration 1 mg/mL, dose 2.5 mg, lockout 10 min) was used. All patients received oral acetaminophen (1,000 mg 4 times a day), starting in the post-anesthesia care unit. For thromboprophylaxis, an injection of 5,000 IE dalteparin was administered subcutaneously for 5 days, starting 8 h postoperatively on the day of surgery. Ondansetron and metoclopramid were used for the treatment of nausea and vomiting. All patients received laxatives. At 48 h postoperatively, patients received controlled-release oxycodone (10 mg twice daily) and immediate-release oxycodone (5–10 mg).

Baseline preoperative values were recorded by the anesthesiologist for each patient. Surgical time was recorded as the interval between skin incision and closure. Supplementary cumulated morphine requirements were recorded for 48 h. Intensity of knee pain was measured both at rest and during coughing (first 4 h after surgery) and at rest and during walking (6–72 h after surgery) on a 0–100 mm VAS (where 0 = no pain, 100 = worst imaginable pain). Assessments of pain, of occurrence of nausea and itching on a 3-point numeric rating scale (1 = mild, 2 = moderate, 3 = severe), and of vomiting and constipation (no bowel function for 72 h) were made by patients themselves every 2 h on the day of surgery and every 4 h on postoperative days 1 to 3, excluding night-time hours. The highest of the 4 VAS scores in each interval was used for further analysis. Urinary retention requiring catheterization (bladder ultrasound showing a volume of more than 350 mL) was recorded. Total time in the post-anesthesia care unit was also recorded. LOS was recorded using standardized discharge criteria. Patients were considered for discharge if sufficient pain relief was obtained (VAS < 30 mm at rest and < 50 during mobilization), if they were able to maintain personal hygiene, if they had a basic mobility score of 6 (e.g. were independent in being able to get in and out of bed, sit and rise from a chair, and walk safely with walking sticks) measured on the cumulated ambulation score (Foss et al. 2006), if they were able to climb stairs, and if there was an uncomplicated wound-healing process, uncomplicated clinical and radiographic outcomes, no evidence of deep vein thrombosis, satisfactory hemoglobin level, and at least 90º of knee flexion.

1 month after surgery, each patient was contacted by phone by the first author (KVA) for information regarding wound complications, infections, and thromboembolic complications.

### Statistics

Calculation of sample size was based on an expected difference of 40% in analgesic consumption. With 80% power (α = 0.05, β = 0.2), a sample size of 36 patients per group would be required. A conservative sample size of 80 was chosen to allow for incomplete data collection.

Data were analyzed using the Mann-Whitney test, Pearson's chi-squared test, or Fisher's exact test. Results are reported as medians with interquartile range (IQR) or as frequencies. A p-value of < 0.05 was considered significant. EpiData, version 3.1 (EpiData Association, Odense, Denmark) was used for data entry, and statistical analysis was performed with STATA software version 10.0 (SPSS Inc., Chicago, IL).

## Results

The protocol was violated regarding sample size. Due to a prolonged inclusion period, it was decided to terminate the study when 40 patients had completed the protocol. From Jan 2007 until March 2008, 49 patients were enrolled in the study; 7 patients (4 patients in group E and 3 patients in group A) were withdrawn before receiving any study medication because of failed spinal anesthesia. 2 patients in group E were excluded from the trial after randomization owing to an overdose of morphine and failed catheter replacement. 40 patients completed the study, all with 30-day follow-up (Figure).

**Figure:**
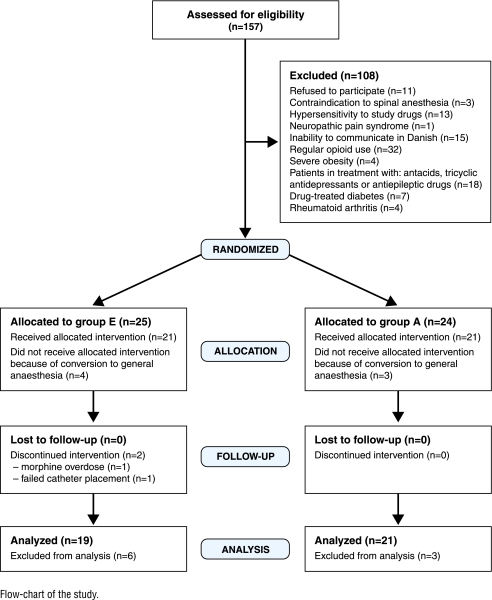
Flow-chart of the study.

Baseline data were similar between groups (Table 1). Time spent in the postanesthesia care unit was reduced by 75 min in group A (with a median time (IQR) of 125 (90–154) min as opposed to 200 (145–275) min in group E (p < 0.001).

**Table 1.** Baseline data

	Group A	Group E
	(n = 21)	(n = 19)
Female / Male (n)	9 / 12	5 / 14
Age, years	67 (63–72)	69 (63–74)
Weight, kg	80 (65–89)	85 (71–88)
BMI	28 (25–32)	28 (26–31)
Smoking, yes/no	6 /15	4 /15
ASA physical status		
I healthy	5	4
II mild systemic disease	16	13
III severe systemic disease	0	2
Duration of surgery, min	119 (94–139)	109 (85–120)

Values are given as frequencies or median with interquartile range in parentheses, where relevant.

Morphine consumption during the active treatment period (0–48 h) was statistically significantly reduced in group A compared to group E (Table 2).

**Table 2.** Morphine consumption, mg

	Group A (n = 21)	Group E (n = 19)	p-value
0–24 h	7.5 (2.5–15)	18 (13–32)	0.05
24–48 h	5 (0–8.8)	15 (5–23)	0.03
0–48 h	11 (3.8–23)	33 (20–40)	0.01

Values are given as median with interquartile range in parentheses. Mann-Whitney U test.

Visual analog pain scores at rest and during mobilization were statistically significantly lower in group A than in group E during the whole study period, with the exception of pain scores during mobilization at 24–48 h after surgery (p = 0.05) (Table 3).

**Table 3.** Pain intensity score (VAS, 0–100 mm) at rest and during walking

	Group A (n = 21)	Group E (n = 19)	p-value
Rest			
2–24	7 (3–25)	30 (10–44)	0.009
24–48	5 (2–21)	33 (9–38)	0.02
48–72	7.5 (2.5–21)	23 (14–42)	0.02
Walking			
2–24	13 (4–41)	37 (12–53)	0.05
24–48	14 (7–35)	41 (27–51)	0.02
48–72	17 (4.5–36)	41 (24–53)	0.02

Values are given as median with interquartile range in parentheses. Mann-Whitney U test.

Most adverse events recorded were found to be similar in the 2 groups. These included episodes of nausea, vomiting, and itching. The intraarticular group (group A) had a significantly lower incidence of constipation (p = 0.004) and shorter time with urinary retention (p = 0.03). No adverse reactions regarding the local anesthetic infiltration and infusion were observed.

The median (IQR) length of stay was 4 (3–5) days in group A and 4 (4–5) in group E; however, discharge criteria were met earlier in group A (3 (3–3.5) days) than in group E (4 (3–5) days) (p < 0.004).

One patient in the intraarticular group developed a deep infection after a spinal abscess, and 2 patients in the epidural group were treated with antibiotics due to wound complications during the 30-day follow-up.

## Discussion

In this study comparing a peri- and intraarticular technique with continuous epidural infusion combined with intravenous ketorolac treatment, we found that the former treatment was associated with an opioid-sparing effect and reduced intensity of pain for 48 h. Time spent in the postanesthesia care unit and days until discharge criteria were fulfilled were shorter in favour of the peri- and intraarticular technique. We did not find any advantage regarding the occurrence of side effects, with the exception of constipation and urinary retention.

Our study has some limitations. Firstly, blinding of patients and caregivers was not attempted. Data collection and analysis were carried out by the first author (KVA) who was not blinded because catheter placement was obvious.

This study was inspired by the local infiltration analgesia technique developed by Kerr and Kohan (2008). Our findings are consistent with the results of 3 recent randomized, controlled trials in knee arthroplasty (Busch et al. 2006, Vendittoli et al. 2006, Toftdahl et al. 2007) in which patients received either peri- or intraarticular treatment or patient-controlled analgesia with opioid or femoral nerve block. All 3 studies showed reduced postoperative pain and opioid requirements.

Patients in this study received 300 mg ropivacaine followed by 384 mg over a 48-hour period. None of the patients reported any toxic symptoms due to local analgesics. In previous studies, infiltration with 400 mg ropivacaine without or combined with one intraarticular injection of 150 mg ropivacaine has shown plasma concentrations far below the toxic level (0.6 μg/mL) (Busch et al. 2006, Vendittoli et al. 2006).

Non-steroidal anti-inflammatory drugs are commonly used analgesics for minor surgery and are useful adjunctive analgesics in patients undergoing major surgery, reducing pain and opioid requirements. To our knowledge, this is the first study that has had adjustment for the systemic effects of intraarticular ketorolac.

2 studies have investigated the effect of continuous intraarticular infusion after TKA (DeWeese et al. 2001, Nechleba et al. 2005). Both of them assessed intraarticular infusion with bupivacaine compared to PCA epidural analgesia and placebo, and they found no or only poor effect. The negative results of these 2 studies are not surprising because of the use of drainage and relatively low analgesic solutions. To our knowledge, there have been no studies investigating the effect of high-volume periarticular infiltration and continuous intraarticular infusion after TKA.

The quality of analgesia provided by the epidural infusion was disappointing. The concept of epidural treatment varies, and there is no “gold standard” to which all other treatment regimes can be compared, and therefore the epidural regime chosen for this study may not be optimal. Epidural analgesia can be improved through the synergistic effect of opioids. However, Andersen et al. (2007a) found no difference in pain intensity scores between peri- and intraarticular injection of ropivacaine and ketorolac compared with continuous epidural infusion of ropivacaine plus morphine in 80 patients after total hip arthroplasty. However, they demonstrated an opioid-sparing effect and reduction in side effects using the intraarticular administration.

There has been concern about the risk of infection with the use of intraarticular or wound catheters after major orthopedic surgery. In this study, 1 patient developed a deep infection after a spinal abscess during the 30-day follow-up. To our knowledge, no studies have had the capacity to investigate the potential risk of infection by intraarticular catheters after TKA. In a metaanalysis of studies involving continuous infusion of local anesthetic into the surgical wound after various surgical procedures, Liu et al. (2006) found that there was no statistically significant difference in wound infection rates between patients who received active treatment (0.7%) and those who received placebo treatment (1.2%). Studies investigating the efficacy of local infiltration analgesia after unicompartmental knee arthroplasty or TKA have not found any increased frequency of infections (Bianconi et al. 2003, Vendittoli et al. 2006, Toftdahl et al. 2007, Essving et al. 2009) but in most studies the follow-up period is often very limited.

In conclusion, the intraarticular technique offers advantages in its simplicity and minimal risk of complications.
